# Predicting Fluency With Language Proficiency, Working Memory, and Directionality in Simultaneous Interpreting

**DOI:** 10.3389/fpsyg.2018.01543

**Published:** 2018-08-21

**Authors:** Yumeng Lin, Qianxi Lv, Junying Liang

**Affiliations:** Department of Linguistics, Zhejiang University, Hangzhou, China

**Keywords:** fluency, working memory, language proficiency, directionality, simultaneous interpreting

## Abstract

Simultaneous interpreting (SI) is a complex bilingual verbal activity that poses great challenges for working memory (WM) and language proficiency. Fluency is one of the crucial indicators in evaluating SI quality, the violation of which is characterized by disfluency indicators such as interruptions, hesitations, repetitions, corrections, and blanks. To uncover factors underlying fluency in SI, 22 interpreting students performed a battery of tasks to test their language proficiency and WM. Two SI tasks, both from Chinese to English and from English to Chinese, were also conducted, and fluency was evaluated according to the five indicators. Two factors (language proficiency and WM) and the five objectively measured disfluency indicators were then used as input for a regression analysis in both directions to model factors underlying fluency in SI performance. The results reveal that, with fluency measured as a whole, WM and directionality yield a significant effect on fluency, and that WM is the only variable that predicts fluency in both directions, accounting for 50 and 51% of the variation in the occurrence of disfluencies in Chinese–English and English–Chinese interpreting, respectively. The findings clarify for the first time the role of language proficiency, WM, and directionality upon fluency in SI, indicating the critical role of WM capability as compared with language skills in fluent production. The research also supports the position that, for interpreting students, interpreting performance tends to be more fluent in the non-native to native language direction.

## Introduction

Simultaneous interpreting (SI) is a highly complex verbal task, requiring listening and comprehension of the source language. It involves the temporary storage and extraction of the meaning (Christoffels et al., 2004, 2006; Tzou et al., 2012; Injoque-Ricle et al., 2015; Aparicio et al., 2017), reformulation of previous information segments into the target language, and the articulation of even earlier segments (Gerver, 1976; Padilla Benitez et al., 1995). Also, cultural nuances and communication rules have to be taken into consideration while retrieving information on how to convey the meaning of the source language (Christoffels et al., 2004; Liang et al., 2018).

Given the extreme demands imposed by SI, working memory (WM) and language proficiency are supposed to be highly predictive of SI performance (Frauenfelder and Schriefers, 1996). Several SI models have been proposed highlighting these two key variables. In two early models, Gerver (1976) and Moser (1978) posit a critical role for WM in SI. Gerver (1976) model emphasizes that different stages of the SI information processing involve a series of temporal storage systems, with WM as an important component. Two buffers work in this model, with the input buffer storing the source text and the output buffer receiving extra monitoring. In Moser (1978) model, WM functions as a component in charge of the storage of processed text units. The source language message is received in the auditory receptor system and is stored in the perceptual auditory storage. The stored verbal information then triggers the search for a conceptual base supported by the concepts stored in long-term memory. Gile’s effort model of SI explains the role of WM and language proficiency in terms of several core efforts involved in SI processes. This model depicts SI as a process involving listening, production, and memory efforts. Sufficient memory capacity and a high level of language proficiency are required for each specific effort, as well as for anticipation, to realize a smooth delivery in interpreting (Gile, 2009).

The mechanism described in these SI processing models may explain why research in this domain is centered on the role of WM and language proficiency. On the one hand, prior studies focusing on WM have mainly investigated two basic questions. First, do training and practice in interpreting enhance WM capacity? Second, is WM related to interpreting performance? Research into the first question has assessed the WM capacity of interpreters with different levels of expertise, such as trained or professional interpreters, bilinguals, and SI students. The results indicate that interpreters with higher level of expertise outperform non-interpreters in WM capacity (e.g., Padilla Benitez et al., 1995; Christoffels et al., 2006; Shen and Liang, 2015; Dong et al., 2018). However, Liu et al. (2004) and Injoque-Ricle et al. (2015) claimed that no significant difference in WM exists across professional interpreters with different length of experience. As for the second question, a significant difference in SI performance between different WM groups has been reported several times, with higher WM contributing to better SI performance (e.g., Padilla Benitez et al., 1995; Tzou et al., 2012; Zhang, 2012; Injoque-Ricle et al., 2015; Macnamara and Conway, 2016).

On the other hand, regarding the role of language proficiency in SI, Tzou et al. (2012) examined the influence of second language proficiency and length of formal training on SI performance and WM. L2 proficiency is reported to be positively associated with better SI performance and higher WM capacity. Blasco Mayor (2015) also demonstrated that L2 listening comprehension proficiency has a significant effect on undergraduate students’ interpreting ability and is therefore a suitable predictor for interpreting aptitude.

Despite the efforts focusing on the role of WM and language proficiency in SI, it is noteworthy that none of these studies investigate this issue by comparing the effects of both factors in a single experiment with the same participants and materials (cf. Tzou et al., 2012). With Tzou et al.’s work, the correlation among all the relevant aspects makes it impossible to determine or discriminate the independent roles of these variables in SI.

Additionally, the above-mentioned studies generally explore the given topic with no regard for interpreting direction, which has been demonstrated to influence SI performance (e.g., Denissenko, 1989; Schweda Nicholson, 1992; Hyönä et al., 1995; Tommola and Helevä, 1998; Seleskovitch, 1999; Chang, 2005; Donovan, 2005). However, no consensus has been reached on the favored direction in SI performance. Some studies have argued for the benefits of interpreting from one’s native language. For example, Denissenko (1989) and Tommola and Helevä (1998) reported that interpreting into the non-dominant language leads to interpretations of higher quality. The advantages in this direction lie in the fact that comprehension of the source text may reduce the loss of crucial information and the occurrence of misinterpretations, thus ensuring higher interpreting quality. By contrast, some extensive surveys suggest that interpreters prefer to interpret into their native language, though it may be a result of unbalanced training experience lengths between two directions (Donovan, 2005; Lim, 2005; Martin, 2005; Nicodemus and Emmorey, 2013; Choi, 2015). Additionally, other behavioral research also reveals that interpretations are of better quality when performed into the native language direction. The authors of those studies emphasize an extra cognitive burden and a decrease in interpreting quality while performing interpreting into the non-native language direction (Schweda Nicholson, 1992; Hyönä et al., 1995; Seleskovitch, 1999; Chang, 2005; Donovan, 2005).

Considering the extreme complexity in SI, Gile (2005) suggested that cognitive load is possibly the most important factor determining directional differences in SI performance. With regard to cognitive load, it has been recognized that interpreters generally work at levels of cognitive load close to saturation, which explains the impaired performance even when no clear problem triggers exist (Gile, 2005, 2009). On the one hand, the amount of processing capacity required for speech comprehension and speech production differs (Deìjean Le Feìal, 2003). On the other hand, the requirements for processing the native and non-native language are also diverse. The interaction of these two aspects results in differences in cognitive load between the two different directions in SI. Hence, we assumed that the effect of WM and language proficiency on SI can be better illustrated via an investigation taking directionality into consideration.

Thus, the present study aims to examine the predictive power of WM and language proficiency on SI performance in both interpreting directions. SI performance *per se* is an integrated concept which is largely subjective and probably too abstract to be defined properly, and few studies in the literature have been able to pin down it objectively. We believe that the existing controversies in previous studies may be partly attributed to this very holistic feature. Indeed, there have been some studies examining the general perception and expectation of interpreting quality, and their results suggest that both the content (accuracy and completeness of information) and the form (fluency of delivery, accent, intonation, and voice quality) matter in the evaluation (Bühler, 1986; Pöchhacker and Zwischenberger, 2010; Yu, 2017). In this regard, fluency is employed in the present study as an observable and specific measure for SI performance. Fluency, as suggested by Yu (2017), is one of the most essential criteria contributing to the quality of SI performance. A global survey on conference interpreting quality conducted by Pöchhacker (2012) showed that fluency is perceived to be very important by 71% of 704 interpreters worldwide and ranks third out of 11 quality criteria in importance.

Yu (2017) examined the correlation between judged fluency and judged accuracy of consecutive interpreting. The results show that judges’ ratings for information and grammatical accuracy are both closely correlated with their ratings for four aspects of fluency. In this regard, successful interpreting performance requires fluency as much as accuracy. Existing studies on SI fluency are mostly centered around describing the features of various disfluency types (Macías, 2006; Xu, 2010; Fu, 2012; Wang and Li, 2015). Regarding disfluency types, Pöchhacker (2001) and Dai (2011) categorize fluency into five factors – pauses, corrections, hesitations, omissions, and blanks.

Empirical studies on the relation between pauses and interpreting directions have gained increasing attention in recent years, yet have yielded inconsistent results. Mead and Twain (2000) reported that students produce more filled pauses (any occurrence of a hesitation interjection) when interpreting into their non-native language than into their native language. By contrast, Fu (2012) argued that although partial differences exist between the two interpreting directions, the evidence is not sufficient to substantiate the difference of the pause occurrences between the two directions.

The above-mentioned findings from different domains highlight the relation between influencing factors and SI performance. Nevertheless, three points of interest remain open for discussion. First, some studies have argued that a strong WM capacity is required for successful SI performance, while other studies point to language proficiency in both languages as being more important. Although great efforts have been made in this area, the factors potentially underpinning SI performance have mostly been investigated separately, and hence the relative predictive power of WM and language proficiency has not yet been weighed. Second, directionality may exert load-related influence on SI performance and interact with levels of WM and language proficiency, but the extent to which it does so remains unclear. Third, previous studies on SI fluency have mainly focused on analyzing one particular disfluency phenomenon by describing its features in a separated manner, the relationship between SI fluency and the above-mentioned factors remains to be systematically explored.

Therefore, the present study aims to investigate the contrastive and combined effects of language proficiency, WM, and directionality on SI fluency. Models will be constructed to predict SI fluency with the potentially relevant factors of WM and proficiency in both directions, in contrast to other studies that investigate these factors separately. We offer a novel perspective by tapping into fluency, which can be directly and objectively measured. This approach was anticipated to minimize the subjectivity of judgments by capturing the knowledge quantitatively.

In this work, we mainly aim to address the following questions:

(1) Does SI fluency differ in English–Chinese (E-C) and Chinese–English (C-E) directions?
(2) Do language proficiency and WM influence SI fluency in both interpreting directions?
(3) How do language proficiency and WM predict fluency in E-C and C-E directions, respectively?
(4) Which variable has a greater predictive power of SI fluency?

The first question is intended to investigate the possible effect of direction on SI fluency, while the second and third questions address the puzzle whether WM or language proficiency has a greater impact on the interpreting performance in terms of fluency in both directions. All three questions are addressed by the predictive models that can integrate the two key factors (language proficiency and WM) while disentangling the independent predictive power of each factor in both directions.

The difference in the predictive power between WM and language proficiency could entail two contrasting possibilities. The first possibility is that WM should be a better contributor than language proficiency to SI fluency. Given that fluency mirrors cognitive load during language production, it is very likely that WM would contribute more to the prediction of SI fluency than language proficiency. The second possibility could be the opposite, since SI delivery is highly constrained by language proficiency, it may significantly impact the performance in terms of fluency.

## Methodology

### Participants

A total of 25 students from key universities in China majoring in translation and interpreting (22 females, three males) participated in the experiment, with ages ranging from 21 to 26 years (*M* = 22.77, *SD* = 1.19). The participants provided their written informed consent before the experiment, and were rewarded with course credits or ¥20. All experimental procedures were approved by the Research Ethics Board of Zhejiang University, and were performed in accordance with the approved guidelines. Two of the participants did not finish two interpretation tasks for personal reasons, and one of them had difficulty interpreting the C-E task. Our final sample consisted of 22 students, all native speakers of Chinese.

### Instruments

#### WM Tasks

WM was measured by a reading span test.

The reading span task was the first task jointly tapping the storage and processing functions of WM, originally created by Daneman and Carpenter (1980); 42 unrelated English sentences were arranged into 12 sets with lengths from two to five sentences and with three sets for each length. These sentences, excluding technical terms, consisted of 13–16 words. As soon as the participants finished reading the first sentence, the next one would be presented. After each set of sentences was presented, participants were required to recall and type in the final words of the sentences in this set in the correct order on a white screen. Then, an increasingly longer set would be presented to participants, and the test ended when all three sets had been answered incorrectly. The highest length at which a subject answered correctly on two out of three sets was taken as the score of the subject’s reading span.

#### Language Proficiency Evaluation

Language proficiency was evaluated by a self-report measure and a behavioral measure.

The self-report data were collected from language history questionnaires adapted from Vaid and Lambert (1979), in which participants were required to provide detailed information on their English language learning and rate their English language level in several aspects, including speaking, listening, reading, and understanding. A seven-point scale with a high degree of differentiation was adopted (1 = very little knowledge and 7 = use it like a native speaker).

The behavioral measure assessed reading speed for English phrases. This measure had been used in previous research (Tzou et al., 2012) for evaluating language proficiency. Participants were required to read 30 idiomatic English phrases presented in a random order on the computer screen and press a key immediately after they had finished reading each phrase. The reading time was automatically recorded by the computer with the accuracy of microsecond. The time taken to read all the phrases was applied to evaluate their language proficiency. A comprehension test was also included to ensure that the participants had properly understood the phrases. Participants who spent less time reading were taken to have relatively higher language proficiency.

The final score of language proficiency was calculated by the sum of the two measurements on a maximum score of 100, evenly split between the questionnaire score and the behavioral task score.

#### SI Tasks

Two 2-min audio files of an equivalent level of difficulty were chosen for the SI task.

The material used in the C-E interpreting task (native to non-native language) was selected from a portion of a speech delivered by former Chinese President Hu Jintao at the welcoming banquet of the Beijing Olympic Games, and the material used in the E-C interpreting task (non-native to native language) was from a portion of a speech delivered by Britain’s Queen Elizabeth II at the banquet of Hu Jintao’s visit to England. To minimize the probable effect of differences in material upon interpreting performance, a series of computational based text difficulty measurement was conducted based on several well-established readability formulas such as Coleman-Liau Readability Score, SMOG Index, and Spache Readability Index, which are viable for both Chinese and English (Liu and Chiu, 2009; McNamara et al., 2010; Akbari and Segers, 2017). The given materials got a SMOG degree between 12 and 14. According to the SMOG conversion table, the SMOG degrees of 12–14 are of the same degree of readability within the SMOG scale, meaning that the material is understandable for middle school students (Mc Laughlin, 1969). No other differences were found in the values of readability indices in the given material. All texts in the materials, both English and Chinese, are thus matched in terms of the text readability level. Moreover, the material selected was suitable for interpreting students and was comparable in these aspects. First, the two audio files were both typical of a welcoming speech. Second, they were selected from the same scenario of a welcoming banquet. Third, the two files had a similar speech duration and a similar average speech rate. Background information was explained beforehand, and no difficult words or expressions were involved in the materials.

#### Fluency Evaluation

The present study replicated the fluency evaluation in Wang (2016), and used five indicators of fluency evaluation: interruption frequency, hesitation frequency, repetition frequency, correction frequency, and blank frequency. Interruptions in SI include interruptions of semantic coherence and interruptions of grammatical structure. Previous studies report that grammatical pauses (occurred at grammatical junctures) longer than 1.4 s and semantic coherence pauses (pauses for message segmentation) longer than 0.56 s can be regarded as harmful pauses, and these are thus labeled as interruptions of disfluency (Duez, 1982; Tissi, 2000). Hesitations refer to expressions such as “uh” and “huh” in SI. Repetitions refer to repeated usage of words, phrases, and sentences in different linguistic units in SI. Correction refers to the revision and supplementation of information that is incorrect or omitted. Blank refers to the omission of information from the source language (a language which is to be translated into another language) to the target language (a language into which another language is to be translated).

The recordings of the interpretations were transcribed by a graduate student. Then, two other graduate students were instructed to mark the different types of disfluencies while listening to the recordings carefully with the software of Adobe Audition 3.0. The marks were assigned according to the rules described above. To guarantee the accuracy of marking, the type of each mark was checked by two researchers in the field of interpreting. When disagreements arose, another expert in interpreting studies was consulted until all disagreements were settled. The numbers of all types of disfluencies were calculated finally according to the checked marks.

### Procedure

All participants were tested in a quiet room. Detailed instructions were given in advance. The reading span test was administered first. Participants were required to recall and type in the final words of the sentences in the correct order. The behavioral language proficiency task was then administered. Participants read 30 English phrases presented on the computer screen, and the reading time was automatically recorded immediately after all phrases had been read. Finally, the SI task was administered. Participants were required to take two SI tasks, including one C-E interpretation and one E-C interpretation, each about 2 min in length. The English speech was played first, followed by the Chinese speech. All the participants were asked to interpret the speeches in the simultaneous mode when their interpretations were recorded. Finally, each participant filled in the questionnaire presented in English.

## Data Analysis and Results

To examine the effect of directionality on SI fluency, we first conducted a paired-samples *t*-test. Then, to investigate to what extent language proficiency and WM can explain the variance in SI fluency in each interpreting direction, a series of linear regression models were built.

For the two potential influencing variables (WM and language proficiency), analyses were conducted in each interpreting direction using two approaches. First, the overall effect on fluency as a whole was tested by using the total frequency of all the five disfluency indicators as the dependent variable. Each disfluency phenomenon reflects the processing difficulty of SI in different aspects. And next, the frequency of each disfluency indicator was applied as the dependent variable, respectively, in order to explore the predictive power of the variables for each disfluency phenomenon.

Before conducting the analysis on the two variables (language proficiency, *M* = 69.5, *SD* = 4.2; WM, *M* = 3.45, *SD* = 0.83) and SI fluency in each interpreting direction, we first present the mean values, SD, and range for the five disfluency indicators in both directions. Descriptive statistics of five types of disfluency frequency in two interpreting directions are displayed in **Table 1**.

**Table 1:** Descriptive statistics of five types of disfluency frequency in two interpreting directions.

	Interruptions		Hesitations		Repetitions		Corrections		Blanks	
					
	E-C	C-E	E-C	C-E	E-C	C-E	E-C	C-E	E-C	C-E
Mean	5.77	12.95	7.32	14.05	1.5	3	2.4	3	1.86	2.77
SD	2.45	5.62	3.66	7.54	1.68	2.65	1.4	1.8	1.49	1.72
Range	2–11	5–27	1–12	2–27	0–6	0–9	0–5	0–6	0–5	0–7


### Effect of Directionality on Fluency in SI

**FIGURE 1:**
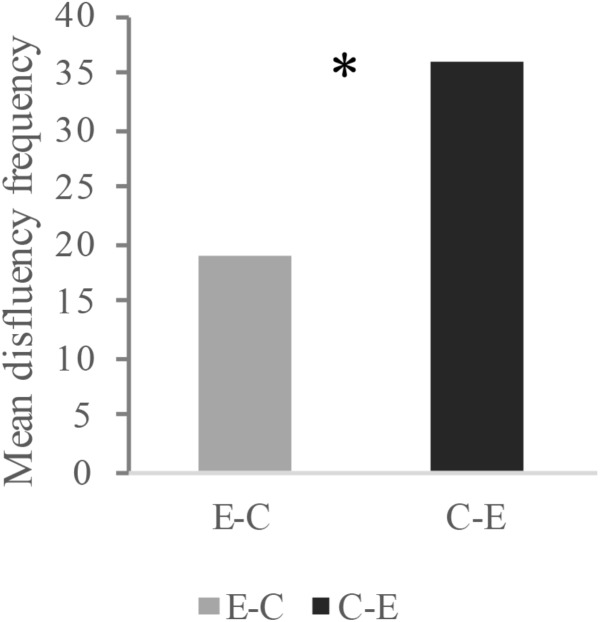
Disfluency frequency of two interpreting directions (average number over the participants). ^∗^Indicates where significant differences exist.

To examine whether interpreting directionality has a significant effect on SI fluency, we first performed a paired-samples *t*-test, in which fluency was measured by the sum frequency of the five disfluency indicators. Preliminary analysis showed that the differences between the data pair were normally distributed, and no outlier was detected. As is shown in **Figure 1**, a significant effect of directionality on SI fluency was observed, *t*(21) = 8.64, *p* < 0.001, *d* = 1.84, indicating that interpreting students performed more fluently in the E-C interpreting direction than in the C-E interpreting direction.

**FIGURE 2:**
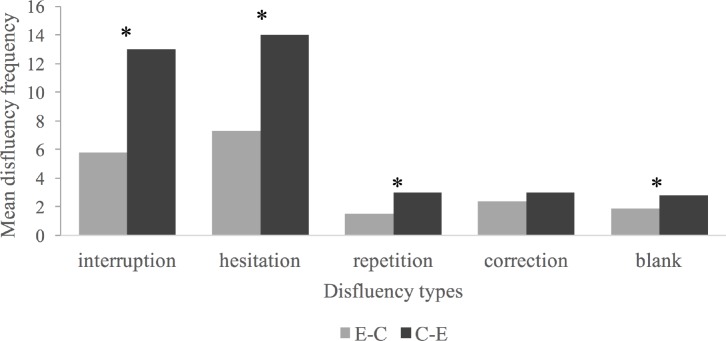
Average frequency of five disfluency indicators in two interpreting directions. ^∗^Indicates where significant differences exist.

In order to further examine whether directionality has a significant effect on each disfluency indicator of SI, we next conducted a series of paired-samples *t*-tests and a Wilcoxon signed-rank test. The differences between the data pairs were screened to identify outliers, which were removed from the data. The difference values of four indicators followed a normal distribution except for the repetition frequency, which was therefore analyzed using a Wilcoxon signed-rank test. The frequencies of interruption, hesitation, correction, and blank in both directions were analyzed by paired-samples *t*-tests. As **Figure 2** illustrates, interpreting directionality was demonstrated to have a significant effect on interruption frequency, *t*(20) = 8.04, *p* < 0.001, *d* = 1.75, hesitation frequency, *t*(21) = 5.63, *p* < 0.001, *d* = 1.20, and blank frequency, *t*(20) = 3.29, *p* = 0.004, *d* = 0.72. The Wilcoxon signed-rank test, using repetition frequency in both directions as dependent variables, showed that the frequency of repetition was significantly higher in the C-E than in the E-C direction, *z* = -3.09, *p* = 0.002, *r* = 0.56. No difference was detected between the two directions in terms of correction frequency (*p* = 0.56).

### Regression Analysis

To investigate whether the two key variables (WM and language proficiency) can predict SI fluency in terms of both E-C and C-E interpreting directions, regression models in both directions were constructed. To rule out the probable confusion in data analysis, the Pearson correlation between WM and language proficiency was conducted. The results indicated no significant correlation between these two variables (*r* = -0.03*, p* = 0.90), thus confirming the validity of regression analysis.

First, regression was conducted using fluency measured by the sum of the five indicators’ frequency as the dependent variable, and WM and language proficiency as the independent variables, with the stepwise method. Residuals were examined to check for homoscedasticity, normality, independence, and linearity, and no multicollinearity was observed (VIF = 1.00, tolerance > 0.1). One outlier of repetition frequency was excluded from the data.

The results revealed that the variable of language proficiency was removed from both the two initial models in the two interpreting directions, and only WM was useful for predicting fluency. Both the two models were statistically significant [*F*_(1,20)_ = 22.11, *p* < 0.001, adjusted *R*^2^ = 0.50, for the E-C direction; *F*_(1,20)_ = 23.07, *p* < 0.001, adjusted *R*^2^ = 0.51, for the C-E direction). The independent variable that contributes to predicting the dependent variable is WM (*β* = -0.73, *t* = -4.70, *p* < 0.001, for the E-C direction; *β* = -0.73, *t* = -4.80, *p* < 0.001, for the C-E direction). The results of these two models are presented in **Tables 2, 3**. The following regression equations using WM capacity to predict disfluency in SI in both directions were obtained:

$$\mathrm{Frequency}\;\mathrm{of}\;\mathrm{disfluency}\;(E-C)\;=\;-6.686^{*}\;\mathrm{WM}\;+\;41.959$$

$$\mathrm{Frequency}\;\mathrm{of}\;\mathrm{disfluency}\;(C-E)\;=\;-11.22^{*}\;\mathrm{WM}\;+\;74.579$$

**Table 2:** Model 1 (dependent variable: frequency of disfluency: E-C).

		*B*	β	Sig.	Adjusted *R*^2^
Model 1	(Constant)	41.959		0.000	0.501
Predictor	WM	-6.686	-0.725	0.000	
Removed variable	Language proficiency		-0.134	0.397	


**Table 3:** Model 2 (dependent variable: frequency of disfluency: C-E).

		*B*	β	Sig.	Adjusted *R*^2^
Model 2	(Constant)	74.579		0.000	0.512
Predictor	WM	-11.220	-0.732	0.000	
Removed variable	Language proficiency		-0.075	0.633	


#### Regressions for the Five Types of Disfluency in the E-C Direction

Next, separate regression analyses of the frequencies of the five disfluency indicators were conducted. Preliminary analysis confirmed the homoscedasticity, independence, and linearity of the data and thus met the assumptions for multiple regression analysis. Residuals of the independent variables were normally distributed. No multicollinearity was observed (VIF = 1.00, tolerance > 0.1).

Stepwise regression was selected as the method of regression analysis for each model. In E-C SI, the first model, using interruption frequency as the dependent variable, was statistically significant, *F*_(2,19)_ = 16.92, *p* < 0.001, adjusted *R*^2^ = 0.60. No variable was removed, and the independent variables that predicted the dependent variable were language proficiency (*β* = -0.56; *t* = -4.10, *p* = 0.001) and WM (*β* = -0.58; *t* = -4.24, *p* < 0.001). Therefore, the following regression equation of interruption frequency in E-C SI, predicted by language proficiency and WM was obtained. The result of this model is presented in **Table 4**.

$$\mathrm{Interruption}\;\mathrm{frequency}\;(E-C)\;=\;34.533\;-\;0.328^{*}\;\mathrm{language}\;\mathrm{proficiency}\;-\;1.722^{*}\;\mathrm{WM}$$

**Table 4:** Model 3 (dependent variable: interruption frequency: E-C).

		*B*	β	Sig.	Adjusted *R*^2^
Model 3	(Constant)	34.533		0.000	0.603
Predictors	WM	-1.722	-0.583	0.000	
	Language proficiency	-0.328	-0.564	0.001	


**FIGURE 3:**
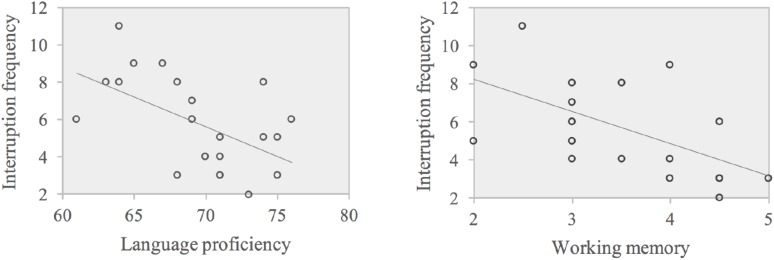
Scatter plots of correlations between WM and interruption frequency (left panel), language proficiency, and interruption frequency (right panel) in E-C.

The absolute value of the standardized regression weighting coefficient was larger for WM than for language proficiency, suggesting that WM contributed more to explain the variance in the interruption frequency in E-C SI compared with language proficiency. The scatter plots of the correlations are displayed in **Figure 3**.

The second model, using hesitation frequency as the dependent variable, was statistically significant, *F*_(1,20)_ = 8.42, *p <* 0.001, adjusted *R*^2^ = 0.26. Language proficiency was removed from the model, and the only predicting variable was WM (*β* = -0.54; *t* = -2.90, *p <* 0.001). Both the variables of language proficiency and WM were removed from the third model, which used repetition frequency as the dependent variable, indicating that neither language proficiency nor WM could predict repetition frequency in the E-C direction. The fourth model, using correction frequency as the dependent variable, was statistically significant, *F*_(2,19)_ = 11.02, *p* = 0.001, adjusted *R*^2^ = 0.49, with no variable removed. As shown in **Table 5**, the predicting variables were language proficiency (*β* = -0.36; *t* = -2.31, *p* = 0.032) and WM (*β* = -0.65; *t* = -4.15, *p* = 0.001). Therefore, the following regression equation of correction frequency in E-C SI, predicted by language proficiency and WM, was obtained:

$$\mathrm{Correction}\;\mathrm{frequency}\;(E-C)\;=\;14.553\;-\;0.12^{*}\;\mathrm{language}\;\mathrm{proficiency}\;-\;1.095^{*}\;\mathrm{WM}$$

**Table 5:** Model 4 (dependent variable: correction frequency: E-C).

		*B*	β	Sig.	Adjusted *R*^2^
Model 4	(Constant)	14.553		0.001	0.488
Predictors	WM	-1.095	-0.648	0.001	
	Language proficiency	-0.120	-0.361	0.032	


**FIGURE 4:**
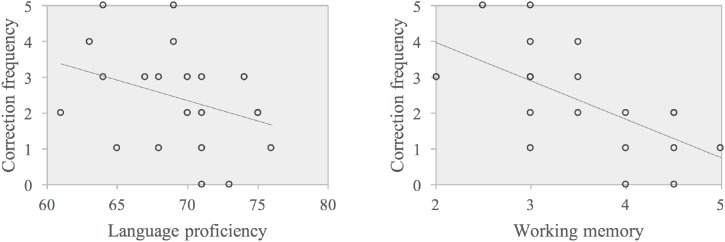
Scatter plots of correlations between WM and correction frequency (left panel), language proficiency, and correction frequency (right panel) in E-C.

The absolute value of the standardized regression weighting coefficient was larger for WM than for language proficiency, suggesting that WM contributed more to explain the variance in the correction frequency in E-C SI compared with language proficiency. The scatter plots of these correlations are displayed in **Figure 4**.

The fifth model, using blank frequency as the dependent variable, was statistically significant, *F*_(1,20)_ = 6.22, *p* = 0.02, adjusted *R*^2^ = 0.20. Language proficiency was removed from this model, and the predicting variable was WM (*β* = -0.49; *t* = -2.49, *p* = 0.02).

#### Regressions for the Five Types of Disfluency in the C-E Direction

Regarding C-E SI, the first model, using interruption frequency as the dependent variable, was statistically significant, *F*_(2,19)_ = 7.28, *p* =0.005, Adjusted *R^2^* = 0.37, and the independent variables that predict interruption were language proficiency (*β* = -0.471, *t* = -2.73, *p* = 0.013) and WM (*β* = -0.473; *t* = -2.74, *p* = 0.013). The result of this model is presented in **Table 6**. The following regression equation of interruption frequency in C-E SI, predicted by language proficiency and WM, was obtained:

$$\mathrm{Interruption}\;\mathrm{frequency}\;(C-E)\;=\;67.78\;-\;0.63^{*}\;\mathrm{language}\;\mathrm{proficiency}\;-\;3.203^{*}\;\mathrm{WM}$$

**Table 6:** Model 5 (dependent variable: interruption frequency: C-E).

		*B*	β	Sig.	Adjusted *R*^2^
Model 5	(Constant)	67.780		0.001	0.374
Predictors	WM	-3.203	-0.473	0.013	
	Language proficiency	-0.630	-0.471	0.013	


**FIGURE 5:**
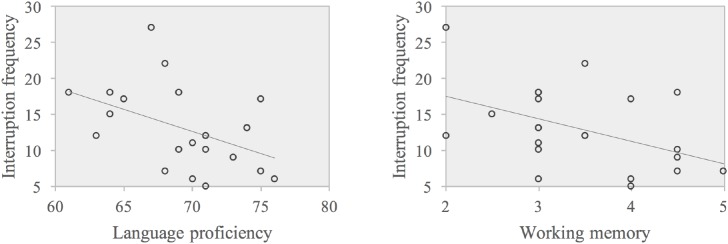
Scatter plots of correlations between WM and interruption frequency (left panel), language proficiency, and interruption frequency (right panel) in C-E.

The absolute value of the standardized regression weighting coefficient was larger for WM than for language proficiency, which was the same as the result in the E-C direction. Therefore, WM showed a greater effect on interruption frequency in both directions. The scatter plots of these correlations are displayed in **Figure 5**.

The second model, using hesitation frequency as the dependent variable, was statistically significant, *F*_(2,19)_ = 14.52, *p* < 0.001, adjusted *R*^2^ = 0.56, with no variable removed. The predicting variables were language proficiency (*β* = 0.34, *t* = 2.34, *p* = 0.03) and WM (*β* = -0.69; *t* = -4.79, *p* < 0.001). The result of this model is presented in **Table 7**. The following regression equation of hesitation frequency in C-E SI, predicted by language proficiency and WM, was obtained:

$$\mathrm{Hesitation}\;\mathrm{frequency}\;(C-E)\;=\;-6.35\;+\;0.606^{*}\;\mathrm{language}\;\mathrm{proficiency}\;-\;6.278^{*}\;\mathrm{WM}$$

**Table 7:** Model 6 (dependent variable: hesitation frequency: C-E).

		*B*	β	Sig.	Adjusted *R*^2^
Model 6	(Constant)	-6.35		0.738	0.563
Predictors	WM	-6.278	-0.691	0.000	
	Language proficiency	0.606	0.338	0.030	


**FIGURE 6:**
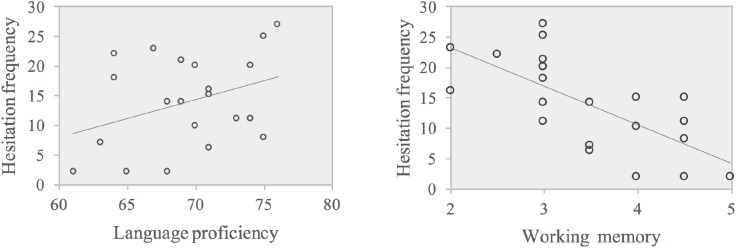
Scatter plots of correlations between WM and hesitation frequency (left panel), language proficiency, and hesitation frequency (right panel) in C-E.

The absolute value of the standardized regression weighting coefficient was larger for WM than for language proficiency. Therefore, WM showed a greater effect on hesitation frequency in the C-E direction. The scatter plots of these correlations are displayed in **Figure 6**.

Both the variables of language proficiency and WM were removed from the third and fourth models, which used repetition and correction frequency as the dependent variable, respectively, indicating that neither language proficiency nor WM could predict repetition and correction frequency in the C-E direction. The last model using blank frequency as the dependent variable was statistically significant, *F*_(1,20)_ = 5.32, *p* = 0.032, adjusted *R*^2^ = 0.17. WM was removed from this model, and the predicting variable was language proficiency (*β* = 0.46, *t* = 2.31, *p* = 0.032). The summary of the regressions’ results is displayed in **Table 8**.

**Table 8:** Summary of the regressions’ results (LP = language proficiency).

	Overall disfluency	Interruption	Hesitation	Repetition	Correction	Blank
Predictors (E-C)	WM	WM	WM		WM	WM
	(β = -0.73)	(β = -0.58)	(β = -0.54)		(β = -0.65)	(β = -0.49)
		LP			LP	
		(β = -0.56)			(β = -0.36)	
Predictors (C-E)	WM	WM	WM			LP
	(β = -0.73)	(β = -0.473)	(β = -0.69)			(β = 0.46)
		LP	LP			
		(β = -0.471)	( β = 0.34)			


## General Discussion

The primary goal of the present study was to examine the effect of language proficiency, WM and directionality on SI fluency by measuring five indicators, namely, interruptions, hesitations, corrections, repetitions, and blanks. To our knowledge, this is the first study in predicting SI fluency that was measured by objective indicators. Moreover, we have quantified the relationships between fluency and the underlying factors (WM, language proficiency) in both directions and thus uncovered the more influential factor in SI fluency.

The results indicate that all three factors have an effect, to various extents, on SI fluency. Directionality and WM are observed to be the influential factors on the overall SI fluency and most SI disfluency indicators. First, directionality yields a prominent effect on all disfluency indicators except correction. In terms of the other four disfluency phenomena, interpreters perform better in E-C than C-E interpreting in this regard. Second, WM is significantly correlated with interruptions and hesitations in both directions, as well as correction and blank in E-C SI. With regard to language proficiency, only the association with interruptions is detected in both directions. Though it affects the correction frequency in the E-C direction, its explanatory power is also lower than that of WM in this respect. Surprisingly, positive correlations of language proficiency level and occurrences of hesitations and blanks in C-E SI are detected.

In this section, we will first compare our findings to previous studies in order to highlight the contribution of the present study in uncovering the factors that affect fluency. We will then discuss the superior explanatory power of WM, as compared with language proficiency, on SI fluency.

### Effect of Directionality on SI Fluency

Our paired-samples *t*-tests show a distinction in interruption, hesitation, repetition, and blank frequencies between the two interpreting directions, which suggests that directionality is a salient factor that influences SI fluency. This supports the stance that, for interpreting learners, interpreting in the non-native to native language direction tends to yield more fluent output.

This finding probably reflects differing demands in terms of the cognitive load of comprehension and the cognitive load of production. Disfluency phenomena, an easily detectable form of quality deterioration in terms of output delivery (cf. output content), may be reflective of insufficient processing capacity. A fluent interpreting output requires a certain amount of processing capacity. If the load exceeds the total available capacity, cognitive saturation arises, and thus disfluency occurs (Gile, 2009). In Gile (2005), two contrasting models are adopted to quantify the load in each interpreting direction with the assumption that the processing capacity required in the native language is less than that in the non-native language. If comprehension imposes fewer requirements on processing capacity than production, then the total processing load is lower in the non-native to native language direction than in the native to non-native language direction. Conversely, if comprehension requires more capacity, then the native to non-native language direction demands less load (Gile, 2005). Consequently, the fact that interpreting students performed better in the E-C (non-native to native language) direction in the present study favors the idea that comprehension takes up less processing capacity in interpreting than production. However, the measure in the present study is based on the output, suggesting that comprehension has not been fully checked. Future studies involving the participants’ comprehension may help deepen our understanding in this respect.

This finding is consistent with previous survey results on interpreters’ preference toward interpreting into the native language (Pavlović, 2007; Nicodemus and Emmorey, 2013; Choi, 2015). The longer training and practical experience may partly account for the interpreters’ preference for the non-native to native language interpreting, because most interpreters are trained more in this direction in accordance with the mainstream practice in large international organizations, such as the United Nations and the European Parliament (Chmiel, 2016). However, in the present study, WM can predict four disfluency indicators in the non-native to native language direction but only two in the native to non-native language direction. It means that cognitive ability may play a different role in the two interpreting directions. Consequently, we have good reasons to believe that the different cognitive loads on the two directions also contribute to the more fluent output into the native language. Given that our study measures fluency objectively, it can be concluded that directionality in SI affects not only the preference of interpreters, but also the fluent production of the target language.

### Effect of Language Proficiency and WM on SI Fluency

In contrast to the preexisting studies in which interpreting performance was evaluated according to a holistic view, the current study assesses interpreting quality using five explicitly suggested fluency indicators. It is noted that interruption, the most frequently occurring disfluency phenomenon, is correlated with language proficiency in both directions, which is consistent with the findings of Tzou et al. (2012) and Blasco Mayor (2015). Their studies both indicate that a higher language proficiency can be an indicator of better interpreting performance, whether by interpreters or by learners. Our result indicates that a higher L2 proficiency level contributes to fluent SI production in terms of reducing interruptions. That is, interpreters with a high proficiency level can avoid interruptions in SI in both directions, which suggests that this most frequently occurring disfluency phenomenon can be improved by enhancing language proficiency. In addition, a higher language proficiency level can be related to a better command of syntactic structures, which enhances online comprehension of the non-native language, thus decreasing the rate of correction in the output. By contrast, in C-E interpreting, students with a higher level of proficiency show no advantage in terms of fluency of production. Some of them even omit or hesitate more than students with a lower level of proficiency. This striking results pattern may be explained by the fact that the participants employed in this study are interpreting students who are not fully fledged in all the interpreting strategies. Therefore, their performance may not be stable. The participants with higher language proficiency in the target language may be impeded by other difficulties such as the failure in memorizing or adopting proper strategies. Consequently, the conventional wisdom that a high level of proficiency guarantees fluent delivery of SI into one’s non-native language may not be completely true and other factors may contribute more to the fluent delivery of native to non-native language SI output.

Our finding that WM shows a significant correlation with SI fluency is echoed by recent studies concerning the impact of WM on interpreting performance. These studies show similar findings, employing different interpreting languages, interpreting types, and levels of expertise of the subjects. Injoque-Ricle et al. (2015) investigated the WM span and SI performance of 30 Spanish-speaking professional English interpreters. The results suggest that interpreters’ ability to store or process information may be supported by WM capacity. Macnamara and Conway (2016) investigated this issue using trainee simultaneous interpreters working from English into American Sign Language, in an effort to study the synergism of WM and the effectiveness of SI. They find WM capacity to be one of the parameters predictive of final SI performance. Yenkimaleki and van Heuven (2017) also investigated the effect of memory training on the quality of interpreting with Farsi-to-English interpreting trainees; their data analysis shows that memory training has a positive effect on improving the quality of SI, and particularly on decreasing the omission rate. Therefore, the findings of these studies converge to suggest that interpreters with high WM capacity perform better on SI tasks, which further indicates that this impact holds regardless of interpreting type, language proficiency, or expertise.

Several factors may account for the positive relation between WM and SI fluency and these factors may lead to different disfluencies. Wang and Li (2015) pointed out that unbalanced split attention in multitasking can also lead to non-smooth delivery in SI. When performing multitasking SI, attention is divided between listening and production tasks, which according to Cowan (2000), is difficult since “interpreters are unlikely to share attention adequately between listening and speaking.” On the one hand, too much attention to listening and analysis may result in insufficient WM capacity for production, in which case interpreters may even halt the ongoing output production, hence resulting in interruptions and hesitations. This is borne out in the fact that WM could predict a fairly large amount of variations in the frequencies of interruption and hesitation in both directions in the present study. On the other hand, too much attention allocated to the search for appropriate expressions may lead to insufficient cognitive resources for listening to the input in the interpreters’ non-native language, which gives rise to blanks in interpreting. The results in the present study also show that only the frequency of blanks in interpreting from one’s non-native to native language is significantly predicted by WM capacity, while no such prediction is found in the opposite direction.

Additionally, we assume that interpreters with higher WM may successfully retain a segment of the syntactic structures of the source text and integrate it with subsequently presented information in the target language (Liang et al., 2017). Conversely, interpreters with lower WM may fail to retain the “current” source text structure or start rendering too early, and consequently they will have to wait for subsequent information to fill the gap or correct the incomplete structure. Thus, interruptions, hesitations, and corrections occur more often with interpreters of lower WM. The deep involvement of WM in SI has been recently proved in a quantitative investigation into the dependency distances of SI as compared to consecutive interpreting. This research results demonstrated that expert SI interpreters generally follow the syntactic structures of the source text and thus yield output dependency distance values closer to those of the input (Liang et al., 2017). Dependency distance, as an index of sentence complexity, is defined as the number of words intervening between two syntactically related words, or the difference between the two in linear position. It can reflect cognitive constraints during various tasks (Liu, 2008; Futrell et al., 2015; Liang et al., 2017; Liu et al., 2017). According to Liang et al. (2017), on the one hand, SI output is highly constrained by the input, as interpreters handle the source speech in speech segments formulated in terms of few words or phrases. On the other hand, the WM load imposed in processing and retaining each chunk of information can be relieved after the chunk is interpreted. Therefore, the higher rate of disfluency occurrences in interpreters with lower WM capacity can be attributed to their stronger tendency to relieve the current load before it can be integrated correctly.

### Predictive Power of Language Proficiency and WM for SI Fluency in Two Interpreting Directions

Another aim of the study was to identify which of the two factors appears to be the better predictor of SI fluency in the C-E and E-C directions. According to the linear regression models in both E-C and C-E directions, it was demonstrated that the contribution of WM is much greater than that of language proficiency in predicting SI fluency, accounting for 50 and 51% in C-E and E-C directions, respectively.

This finding suggests that the delivery of SI output is constrained by WM storage and coordination capacity. This is in line with the research of Plevoets and Defrancq (2016) and Mizuno (2017). Plevoets and Defrancq (2016) explore the effect of cognitive load on disfluencies in interpreting, and their results indirectly support the hypothesis that different efforts compete for the same cognitive resources. Mizuno (2017) explored cognitive constraints and their effect on SI performance based on the WM theory (Cowan, 2000), and elucidated how interpreters make use of limited WM capacity to achieve the feat of SI. The findings largely corroborate the hypothesis that when interpreters hold information surpassing the capacity of the focus of attention in SI, disfluencies, errors, or infelicities may occur. The occurrence of correction in SI, for instance, can presumably be attributed to the delay of processing in SI, which increases the number of the items to be held in WM. Given that cognitive load depends on the duration of attentional capture, the ratio of processing time to the total time allowed to perform these tasks becomes a proxy for cognitive load (Barrouillet and Camos, 2012). The structures with differing orders in the source and target languages stay longer in the focus of attention for processing while new chunks also come into the storage. It is assumed that interpreters with intermediate WM capacity may fail to retain the delayed chunks until they can be integrated with later chunks to form a complete output. Instead, he or she may render every chunk consecutively according to the source speech and then return to make corrections after finding that the structure does not fit into the target language.

As suggested in the enlarged embedded process model for SI, Mizuno (2005) emphasized the central executive structure as an indispensable component of the language processing system. Attention switching, coordination of tasks, and the capacity of information storage contribute more to the success of SI than basic language proficiency. Poor coordination and low capacity for information storage are more likely to cause interference and the degradation of efficiency and behavior.

Admittedly, limitations of the present study are still here. First, the transferability of our results on interpreting students to professional interpreters cannot be assumed. Trainees may not have fully developed their language skills, and they may thus use different strategies from professionals. Further research on professional interpreters is needed to complement the current results. Second, we only examined directionality between Chinese and English. More language pairs, as well as more diversified methods, shall be taken into consideration in future studies. Third, since WM and language proficiency cannot fully predict SI fluency, other influencing factors need to be further explored.

## Conclusion

The present study explored the predictive power of language proficiency, WM, and directionality on fluency in SI by investigating 22 interpreting students. The results demonstrate that, first, for interpreting students, interpreting performance tends to be more fluent in the non-native to native language direction. Second, in both E-C and C-E directions, the contribution of WM to SI fluency is much greater than that of language proficiency on the whole.

Taken together, these findings reveal that language proficiency, WM, and directionality all have an effect on fluency in SI. Notably, directionality is observed to be a salient factor that influences SI fluency. It is shown that WM alone can positively predict SI fluency, accounting for 50 and 51% of the variance in the frequency of disfluencies in E-C and C-E interpreting, respectively. WM storage, along with coordination and attention splitting, can predict a large part of the variation in fluency in SI. Finally, our findings on the predictive power of influencing factors on SI fluency may shed light on the selection and assessment of simultaneous interpreters. In addition, in SI training, more effort needs to be attached to the development of interpreting strategies in accordance with specific fluency problems and different interpreting directions. Furthermore, the results may also have practical implications for artificial intelligence. A critical difference between machines and human beings lies in the coordination of cognitive resources, and thus it is possible that a neural net algorithm could be developed to maximally simulate actual human cognition.

## Ethics Statement

This study was approved by the Research Ethics Board of Zhejiang University and granting agency, and was performed in accordance with the relevant guidelines and regulations.

## Author Contributions

YL, QL, and JL conceived and designed the experiments, performed the experiments, collected the data, and performed the data analyses. All authors contributed to the interpretation of results and the writing of the manuscript and approved the final version of the manuscript for submission.

## Conflict of Interest Statement

The authors declare that the research was conducted in the absence of any commercial or financial relationships that could be construed as a potential conflict of interest.
